# Mouth Opened with Tongue Extended (MOTE) maneuver: improvement in tomographic T-staging accuracy of oral cavity cancer: a prospective cross-sectional study

**DOI:** 10.1590/1516-3180.2025.0006.R1.04112025

**Published:** 2026-01-09

**Authors:** Gustavo de Francisco Campos, Ula Lindoso Passos, Renato Sartori de Carvalho, Lucimara Sonja Villela de Miranda, Antônio Marcos Coldibelli Francisco, Miriam Harumi Tsunemi, Cauê Ocaña Demarqui, Daniel Cesar Shirane, Luciana Brito Corrêa, Carlos Neutzling Lehn, Marcelo Fernando Matielo

**Affiliations:** I Departamento de Radiologia e Diagnóstico por Imagem, Instituto de Assistência Médica ao Servidor Público Estadual (IAMSPE), São Paulo (SP), Brazil.; II Departamento de Radiologia e Diagnóstico por Imagem, Instituto de Assistência Médica ao Servidor Público Estadual (IAMSPE), São Paulo (SP), Brazil.; III Departamento de Radiologia e Diagnóstico por Imagem, Instituto de Assistência Médica ao Servidor Público Estadual (IAMSPE), São Paulo (SP), Brazil.; IV Departamento de Patologia, Instituto de Assistência Médica ao Servidor Público Estadual (IAMSPE), São Paulo (SP), Brazil.; V Departamento de Ginecologia, Hospital das Clínicas Samuel Libânio, Pouso Alegre (MG), Brazil.; VI Departamento de Biodiversidade e Bioestatística, Universidade Estadual Paulista “Júlio de Mesquita Filho” (Unesp), Botucatu (SP), Brazil.; VII Departamento de Cirurgia de Cabeça e Pescoço, Instituto de Assistência Médica ao Servidor Público Estadual (IAMSPE), São Paulo (SP), Brazil.; VIII Departamento de Cirurgia de Cabeça e Pescoço, Instituto de Assistência Médica ao Servidor Público Estadual (IAMSPE), São Paulo (SP), Brazil.; IX Departamento de Cirurgia de Cabeça e Pescoço, Instituto de Assistência Médica ao Servidor Público Estadual (IAMSPE), São Paulo (SP), Brazil.; X Departamento de Cirurgia de Cabeça e Pescoço, Instituto de Assistência Médica ao Servidor Público Estadual (IAMSPE), São Paulo (SP), Brazil.; XI Departamento de Cirurgia Vascular, Instituto de Assistência Médica ao Servidor Público Estadual (IAMSPE), São Paulo (SP), Brazil.

**Keywords:** Mouth neoplasms, Diagnostic imaging, Multidetector computed tomography, Neoplasm staging, Carcinoma, squamous cell, Head and neck oncology, Dynamic imaging techniques, Preoperative imaging, Tumor boundary delineation, Radiologic measurement accuracy, Oral radiology

## Abstract

**BACKGROUND:**

Accurate T-staging of oral squamous cell carcinoma (SCC) is essential for surgical planning and prognosis. However, conventional computed tomography (CT) may underestimate tumor extent, particularly when performed without dynamic maneuvers.

**OBJECTIVES:**

To evaluate the accuracy of CT in predicting the T-staging of primary SCC of the oral cavity by comparing scans in the neutral position and with the mouth open and tongue extended (MOTE) maneuver.

**METHODS:**

This prospective cross-sectional study analyzed patients with oral SCC who underwent CT in both positions. Two blinded head-and-neck radiologists measured tumor size and depth of invasion (DOI). An anatomopathological study served as the reference. The accuracy of classifying early and advanced T-stage tumors was determined using diagnostic tests.

**RESULTS:**

Twenty-five patients (72% male, mean age 65.6 ± 11.3 years) were included. Tumor detection sensitivity increased from 72% (95% CI: 51.9–86.9) in the neutral position to 100% (95% CI: 83.4–100) with the MOTE maneuver. Correct T-staging prediction improved from 52–56% in the neutral position to 7276% with MOTE. Accuracy for early-stage (T1/T2) classification rose from 60.0% (95% CI: 39.3–78.1) to 88.0% (95% CI: 68.7–97.4, h = 0.66). Lesion size overestimation decreased from 20.9–23.7% to 15.3–16.1% (p < 0.05), whereas DOI differences were not significant (p > 0.05).

**CONCLUSIONS:**

The MOTE maneuver significantly improved both the sensitivity and accuracy of CT in the preoperative T-staging of oral cavity SCC. Its incorporation into diagnostic protocols may enhance lesion detection and staging reliability in daily clinical practice.

## INTRODUCTION

 Head and neck cancer was the seventh most common cancer worldwide in 2020,^1^ 90% of which were squamous cell carcinomas (SCC). Among these, tumors of the oral cavity are the most frequent subtype and have the highest mortality rate. According to global data, 177,757 deaths from oral cavity cancer occurred in 2020,^2^ corresponding to 1.8% of all cancer deaths,^2^ with an average 5-year survival rate of approximately 50%.^1^

 There has been an increase in the incidence of head and neck cancer in recent years in both developed and underdeveloped countries.^1^ Known risk factors include alcohol and tobacco use, as well as betel quid chewing in Southeast Asia.1 These tumors are more frequent in men and usually affect patients older than 50 years.^3^

 The TNM staging system from the American Joint Committee on Cancer (AJCC) is the most widely accepted and used system, and it allows universal standardization.^3^ The classification considers the tumor in relation to its locoregional extension (T), lymph node involvement (N), and presence of distant metastasis (M), thereby allowing a lesion to be classified without ambiguity.^4^ Since its 8th edition, released in 2017,^4^ the parameter of *depth of invasion* (DOI) has been incorporated into the T-stage definition, recognizing its strong prognostic value.^5^

 Depth of invasion (DOI) directly influences surgical planning, as the National Comprehensive Cancer Network (NCCN) recommends neck dissection in early-stage cT1–T2 N0 tumors with DOI > 0.3 cm.^6^

 Anatomopathological measurements remain the gold standard,^4^ but are only available postoperatively. Therefore, a reliable preoperative imaging method is essential for guiding treatment decisions and avoiding both under- and overtreatment. 

 Computed tomography (CT) is the preoperative imaging study of choice for evaluating the primary lesion and lymph nodes.^3^ However, its accuracy may be limited by metallic dental artifacts and mucosal overlap in the neutral position. Several maneuvers have been proposed to improve visualization, such as the "puffed cheek" and open-mouth techniques.^7-11^

 Maneuvers are also used to assess other organs using CT. The phonation maneuver is useful for evaluating laryngeal tumors or lesions affecting the course of the recurrent laryngeal nerve,^12^ as it evaluates the mobility of the vocal cords. In assessing the hypopharynx, the Valsalva maneuver distends the pyriform sinuses, which usually have the overlying mucosa in a neutral position.^13,14^

 The present study aimed to evaluate the accuracy of CT in the neutral position and with the MOTE maneuver in predicting the presurgical T-staging of oral cavity tumors. 

## MATERIALS AND METHODS

 This was a prospective cross-sectional study in which a comparative analysis of DOI measurements and the size of the lesions obtained on the CT scan was conducted. The presurgical T-staging determined by CT was compared with that obtained from the postsurgical anatomopathological study. 

 The study followed all ethical recommendations and was registered and approved by the hospital’s Research Ethics Committee (CAAE 12810719.8.0000.5463; approval number 3.463.777). Patients were informed about all steps, purposes, potential risks, and benefits of participating in the research and signed a Free and Informed Consent (FIC) form. 

### Study design

 The examinations were performed on a BrightSpeed Elite GE ^®^ multidetector CT scanner with 16 channels by two trained radiology technicians. Patients who attended the radiology service after fasting for 4 hours were instructed to remove mobile dental prostheses and were then positioned in dorsal decubitus on the CT scanner. Acquisitions were performed in the axial plane with 1.25 mm slice thickness after intravenous injection of Henetix^®^ hyposmolar nonionic iodinated contrast medium at a dose of 1.5 ml/kg. 

 Image acquisition began 60 seconds after the injection of iodinated contrast medium. Initially, images were acquired in a neutral position, in which the patient was instructed to relax the facial muscles, keep the mouth closed, and breathe calmly. The images were then acquired using the MOTE maneuver described by Bron et al.,^9^ in which the patient kept the mouth open, extended his tongue out of the oral cavity, and breathed calmly. 

**Figure 1:**
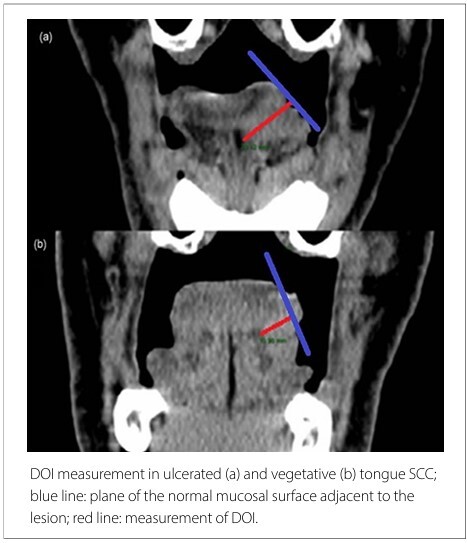
Coronal reformation of the oral cavity.

 The CT images were transferred to the Carestream^®^ system and analyzed by two radiologists, Head and Neck imaging specialists with more than 20 years of experience - named Evaluator 1 (Ev1) and Evaluator 2 (Ev2) – separately and without interobserver communication. Each evaluator measured lesion size and DOI by analyzing the obtained images. As described by Weimar et al.,^15^ DOI was measured perpendicular to an imaginary line drawn along the plane of the normal mucosal surface adjacent to the lesion (**Figure 1**). 

 The intraclass correlation coefficient (ICC) was calculated^16^ to assess the agreement between Ev1 and Ev2 for measuring lesion size and DOI on CT. 

 Once lesion measurements were obtained, tomographic T-staging was determined independently by each evaluator for each patient. Values for tomographic T-staging were based on the TNM 8th edition of the American Joint Committee on Cancer (AJCC).^4^

 After measurements and tomographic T-staging, the patients underwent surgical procedures performed by the institution’s head and neck surgery team. The lesion was resected, the tissue was fixed in 10% formaldehyde and paraffin, and the histological sections were stained with hematoxylin and eosin. An anatomopathological analysis was performed 24 hours after fixation. The interval between CT and the surgical procedure did not exceed 44 days. 

 The size and DOI of the lesion were measured by the same pathologist in all cases. To measure the size of each lesion, the maximum extent observed in macroscopic specimens was considered. For the DOI, the greatest depth of the tumor measured across all the microscopic sections of the specimen was considered. DOI measurements were performed perpendicularly to an imaginary line drawn between the normal basement membranes adjacent to the lesion, as recommended by the College of American Pathologists (CAP).^17^

 Statistical analysis and comparison of lesion size and DOI measured on CT by Ev1 and Ev2 with those obtained in the anatomopathological examination were performed. The mean measurements of each evaluator were determined for each maneuver separately, along with the 95% confidence interval (95% CI) and the percentage of overestimation compared with the anatomopathological values. The Shapiro-Wilk normality test and t-test or Wilcoxon test were used. 

 Once the measurements of the lesions were obtained, T-staging was determined based on the anatomopathological study of each patient, which served as the reference for the T-staging obtained from tomographic measurements. The 8th edition of the AJCC TNM system was also used.^4^

 Tumor T-staging on CT in the neutral position and with the MOTE maneuver was compared with anatomopathology to determine the correct T-staging prediction. 

 The accuracy of the correct classification of early T-stage (T1 and T2) versus advanced T-stage (T3 and T4) tumors was also determined. Preoperative test accuracy was described in terms of sensitivity, specificity, positive predictive value (PPV), and negative predictive value (NPV), expressed as percentages, and 95% confidence intervals (CI) were also calculated. These parameters were determined using diagnostic tests. 

## RESULTS

### Participants

 Initially, 40 patients with SCC of the oral cavity diagnosed at the Head and Neck Surgery Service between July 2019 and July 2021 were selected. Patients with a recurrent lesion (1), unresectable tumors (3), lesions with compromised margins on anatomopathology (3), oropharyngeal tumors (4), or other histological types (4) were excluded. Ultimately, 25 patients were included in the study. 

### Population characteristics

 Of these 25 patients, 18 (72%) were men. The mean age was 65.6 years, with a standard deviation of 11.25. The mean interval between CT and surgery was 9.16 days (standard deviation, 9.65). 

 The most frequent tumor subsite was the oral tongue (14), followed by the floor of the mouth (8) and the hard palate (3). 

### Sensitivity of tumor detection

 The sensitivity to detect the lesion on CT in the neutral position was 72% (18/25, 95% CI: 51.9–86.9) and 100% (25/25, 95% CI:83.4–100) with the MOTE maneuver. This difference corresponded to a large effect size (*h* = 1.12). Importantly, the MOTE maneuver completely eliminated metallic beam-hardening artifacts in all cases, thereby improving lesion conspicuity. 

### Accuracy of tomographic T-staging

 The agreement of DOI measurements in the neutral position performed by Ev1 and Ev2 on CT was considered excellent,^16^ with an ICC > 0.964. 

**Figure 2:**
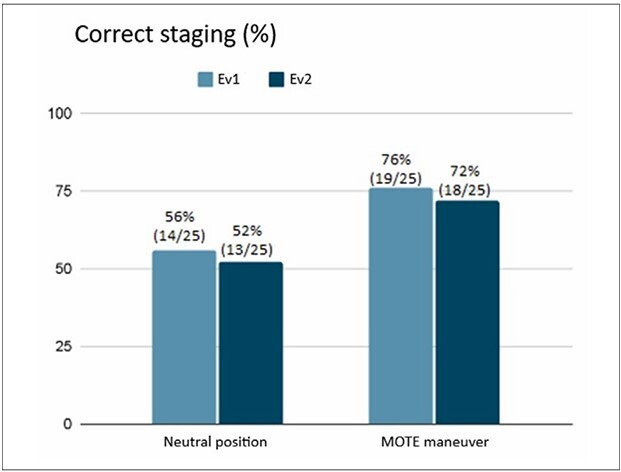
Correct prediction of tomographic T-staging with MOTE maneuver, according to each evaluator in relation to the anatomopathological T-staging of the surgical specimen.

 Using anatomopathological T-staging as a reference, CT in the neutral position correctly predicted the T-staging in 52–56.0% (95% CI: 33.5–77.4) of the cases and the MOTE maneuver in 72–76% (95% CI: 51.5–90) (**Figure 2**). 

 In the neutral position, tomographic classification of early T-stage tumors (T1 and T2) showed a sensitivity of 44.4%, specificity of 100%, PPV of 100%, NPV of 41.1%, and accuracy of 60% (95% CI: 39.3–78.1) (**Table 1**). 

**Table 1:** Distribution of the number of cases to calculate the accuracy in classifying early T-stage tumors (T1 and T2) using CT in the neutral position

	**pT1 e pT2**	**≠ pT1 e pT2**	**Total**
**T1 e T2**	8	0	8
**≠ T1 e T2**	10	7	17
**Total**	18	7	25

T1 e T2: tomographic staging T1 e T2; ≠ T1 e T2: tomographic staging Tx, T3 e T4; pT1 e pT2: pathological staging T1 e T2; ≠ pT1 e pT2: pathological staging T3 e T4.

 Using the MOTE maneuver, differentiation of early T-stage tumors (T1 and T2) from advanced T-stage tumors (T3 and T4) was accurate for both Ev1 and Ev2, as 15 of the 18 pT1/T2 tumors were correctly T-staged on CT. Sensitivity, specificity, PPV, NPV, and accuracy for early T-stage tumor detection were 83.3% (95% CI:58.5–96.4%), 100% (95% CI: 59–100%), 100% (95% CI: 78.2–100%), 70% (95% CI: 45.3–86.7%) and 88.0% (95% CI: 68.7–97.4%), with an effect size of *h* = 0.66. All 7 pT3/T4 tumors (100%) were correctly staged (**Table 2**). 

**Table 2:** Distribution of the number of cases to calculate the accuracy in classifying early T-stage tumors (T1 and T2) using CT with MOTE maneuver

	**pT1 e pT2**	**≠ pT1 e pT2**	**Total**
**T1 e T2**	15	0	15
**≠ T1 e T2**	3	7	10
**Total**	18	7	25

T1 e T2: tomographic staging T1 e T2; ≠ T1 e T2: tomographic staging Tx, T3 e T4; pT1 e pT2: pathological staging T1 e T2; ≠ pT1 e pT2: pathological staging T3 e T4.

 The disagreement between the staging classified by CT and the surgical specimen in formaldehyde led to a comparison of the measurements of the lesions and the determination of the percentage differences. **Table 3** shows the measurements of the lesion size performed by Ev1 and Ev2 and the percentage of overestimation in comparison with the anatomopathological measurements. **Table 4** presents the DOI data. 

**Table 3:** Comparison of the measurements (cm) of the size obtained in the anatomopathological study with the CT measurements (cm) performed by Evaluator 1 (Ev1) and Evaluator 2 (Ev2) in neutral position and with MOTE maneuver

**Size**	**Measurement (cm)**	**p**	**Overestimation (%)**
**Anatomopathological**	2.48 ± 0.41	-	-
**Neutral Ev1**	3 ± 0.58	0.01	20.96
**Neutral Ev2**	3.07 ± 0.62	0.011	23.79
**MOTE Ev1**	2.88 ± 0.49	0.001	16.12
**MOTE Ev2**	2.86 ± 0.5	0.006	15.32

Mean ± 1,96; * SE and t-test/Wilcoxon p value.

**Table 4:** Comparison of measurements (cm) of depth of invasion (DOI) obtained in the anatomopathological study with CT measurements (cm) performed by Evaluator 1 (Ev1) and Evaluator 2 (Ev2) in neutral position and with MOTE maneuver

**DOI**	**Measurement (cm)**	**p**	**Overestimation (%)**
**Anatomopathological**	1.03 ± 0.26	-	-
**Neutral Ev1**	1.32 ± 0.42	0.288	28.15
**Neutral Ev2**	1.37 ± 0.6	1	33
**MOTE Ev1**	1.14 ± 0.33	0.116	10.67
**MOTE Ev2**	1.09 ± 0.31	0.554	5.82

Mean ± 1,96; * SE and t-test/Wilcoxon p value.

 It was found that the size of the lesions was overestimated on CT compared with anatomopathological measurements (p < 0.05). In contrast, although DOI measurements also tended to be overestimated on CT, this difference was not statistically significant (p > 0.05; d = 0.13, indicating a negligible effect size). 

## DISCUSSION

 Relatively simple maneuvers in imaging examinations have been developed since the beginning of the 20th century to increase the sensitivity of lesion detection, reduce artifacts, and perform adequate pretreatment T-staging. 

 Jonsson^13^ reported in 1934 that the modified Valsalva maneuver provided greater sensitivity for detecting lesions in the hypopharynx on contrast radiography. Later, this technique was named the "trumpet maneuver" by Hillel et al. in 1989,^14^ confirming its usefulness for evaluating the piriform sinuses in hypopharyngeal tumors. 

 In 1981, Gamsu et al.^12^ showed that laryngeal structures were better assessed when performing phonation maneuvers during CT scans, favoring the detection of lesions at this site. 

 For better identification of lesions in the oral cavity, Weissman et al.^7^ described the puffed-cheek maneuver on CT scans in 2001, which remains the most accurate technique for evaluating lesions in the buccal mucosa and retromolar trigone. Several studies have shown the benefits of this maneuver, which allows a better assessment of tumor dimensions and their extension.^10,11^

 Henrot et al.^8^ described in 2003 the open-mouth maneuver on CT scans to reduce dental amalgam artifacts in the oral cavity and oropharynx. 

 The MOTE maneuver was described in 2019 by Bron et al.,^9^ who reported a higher rate of correct staging with this maneuver (83%) compared with conventional CT (68%). 

**Figure 3:**
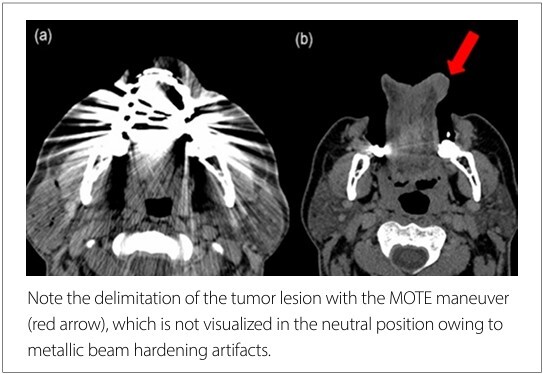
Contrast-enhanced CT in the axial plane in neutral position (a) and with MOTE maneuver (b).

 In our study, CT using the MOTE maneuver demonstrated 100% sensitivity in detecting tumor lesions in the oral cavity and increased the accuracy of presurgical T-staging from 60–88% when compared with the neutral position. We observed better delimitation of the tumor, and metallic beam-hardening artifacts of dental origin were excluded in all cases using this maneuver (**Figure 3**). 

 In our study, the measurements of the tumor lesions on CT were larger than those obtained from formalized specimens. Other authors have also found differences between T-staging on imaging and in surgical specimens, as shown by Kreppel et al. (2016).^18^

 They observed a T-staging agreement of 62%, with the majority of discordant cases (58%) being overstaged. 

 A justification for the difference in the measurements of the lesions on CT and in the anatomopathological study is probably the shrinkage of the piece when soaked in formaldehyde. The overestimation of lesion size with the MOTE maneuver was 16.1% (Ev1) and 15.3% (Ev2), which was lower than in the neutral position (20.9–23.7%). Our data also demonstrated that measurements obtained using the MOTE maneuver were closer to those obtained pathologically. 

 Some authors have studied the reduction in the size of fresh surgical specimens compared with their size after fixation in formaldehyde. The findings of Pangare et al.^19^ and Umstattd et al.^20^ pointed out a reduction in lesion size, ranging from 10.7–23.9%, after 24 hours of fixation in 10% formaldehyde. In our study, lesion size measured on CT showed a reduction ranging from 15.3–16.1% after fixation in formaldehyde, values similar to those reported in these studies, which compared fresh specimens with the same specimens after fixation. 

 Another important observation is that CT detects lesions in living tissue with preserved architecture, which is altered after surgical resection. This may lead to discrepancies in measurements and, consequently, in T-staging. 

 Studies have found overstaging of lesions on CT compared with anatomopathological findings in tumors from various anatomical sites, not only the oral cavity. Winiker et al.^21^ described the overestimation of the lesion dimensions of esophageal tumors on CT and ultrasonography compared to pathology in 2018, with correct T-staging in only 51% of cases, overstaging in 25.5%, and understaging in 23.5%. 

 A relevant factor in therapeutic planning is the correct prediction of DOI using presurgical imaging. DOI is an important predictor of occult lymph node metastasis, and neck dissection is recommended for early-stage cT1–T2 N0 tumors when the DOI is greater than 0.3 cm, according to the NCCN protocol.^6^ In our research, the overestimation of T-staging occurred due to the discrepancy between CT and anatomopathological measurement of lesion size, as the DOI measurements did not show a significant difference between these methods (p > 0.05). The correlation between DOI measurements obtained during imaging and anatomopathological examinations is controversial. Some authors have reported satisfactory correlation between methods, such as Chin et al.,^22^ which is consistent with our study. However, a systematic review and meta-analysis by Li et al.^23^ demonstrated that magnetic resonance imaging tends to overestimate DOI measurements compared with anatomopathological studies; however, this meta-analysis did not evaluate the use of maneuvers. 

 The NCCN recommendation to perform neck dissection in early-stage cT1-T2 N0 tumors with a DOI greater than 0.3 cm^6^ also motivated us to determine the accuracy of CT in correctly differentiating early T-stage (T1/T2) tumors from advanced T-stages (T3 and T4). In our study, we observed an accuracy of 88.0% for this classification using the MOTE maneuver, whereas in the neutral position, the accuracy was 60%. 

 The technique for measuring DOI in pathology consists of drawing an imaginary line between the normal basement membranes adjacent to the tumor and measuring the depth of the lesion from this line.^16^ A challenge in measuring DOI on CT is that the basement membrane is not visualized, as the thickness of the oral mucosal epithelium is < 0.5 cm.^15^ Weimar et al. proposed measuring DOI on CT from the plane of the mucosal surface adjacent to the tumor.^15^ This technique was used in our study for DOI measurement. 

 Our study differs from previous research in the literature. Some prospective studies evaluated the benefits of the puffed-cheek maneuver in imaging examinations of patients with oral cavity tumors and observed improved tumor detection and delimitation; however, they compared imaging examinations with and without the puffed-cheek maneuver, without comparison to anatomopathological measurements. Weissman^7^ and Martínez^10^ studied 7 and 62 patients, respectively, using CT, while Chang^24^ studied this maneuver in 22 patients using PET/CT. 

 Bron et al.^9^ reported greater accuracy in tomographic staging of oral cavity and oropharynx tumors with the MOTE maneuver (83%) compared with conventional CT (68%) in 58 patients. However, that study differs from ours because it was retrospective and included oropharyngeal tumors in addition to oral cavity tumors. 

 Our research differs from these studies because it is prospective and directly compares lesion size and DOI, as well as the accuracy of tomographic T-staging of oral cavity tumors, with anatomopathological findings. We also evaluated the percentage of overestimation of measurements compared with the anatomopathological study 

 The MOTE maneuver is simple, noninvasive, and requires no additional equipment. It reduces metal artifacts, enhances tumorto-soft-tissue contrast, and improves preoperative staging accuracy. Given its feasibility, we recommend incorporating it into the routine CT protocol for patients with suspected or confirmed oral cavity SCC, while considering the approximately 15.3–16.1% overestimation in lesion size compared with anatomopathological measurements observed with this maneuver. 

 We suggest that future studies with larger samples may help determine a measurement conversion coefficient that considers statistically consistent differences between the methods, or determine whether the cutoff points for T-staging measures on CT should differ from those obtained from anatomopathological examination for the T classification within TNM staging. 

## CONCLUSIONS

 The MOTE maneuver significantly enhanced CT performance in the preoperative evaluation of oral cavity SCC, providing higher sensitivity, improved T-staging accuracy, and closer agreement with pathological measurements. This maneuver reduced measurement overestimation and eliminated dental metallic artifacts, resulting in clearer visualization of lesion boundaries. Because of its simplicity and reproducibility, the MOTE maneuver should be incorporated into standard CT protocols for oral cavity tumors, as it offers practical clinical benefits for diagnosis, staging, and surgical planning. 

## Data Availability

The data supporting the findings of this study are available from the corresponding author, Gustavo de Francisco Campos, upon request. The data are not publicly available due to privacy and ethical restrictions.
